# A non‐inferiority analysis of hemoglobin levels in postpartum IUD users in Bangladesh

**DOI:** 10.1002/ijgo.14143

**Published:** 2022-03-06

**Authors:** Suzanna Bright, Georgia R. Gore‐Langton, Parveen Fatima, Farhana Dewan, Afroja Yesmin, Anita Makins

**Affiliations:** ^1^ International Federation of Gynecology and Obstetrics (FIGO) London UK; ^2^ London School of Hygiene & Tropical Medicine (LSHTM) London UK; ^3^ Obstetric and Gynecological Society of Bangladesh (OGSB) Dhaka Bangladesh; ^4^ Oxford University Hospitals NHS Foundation Trust Oxford UK; ^5^ Nuffield Department of Women's & Reproductive Health University of Oxford Oxford UK

**Keywords:** anemia, contraception, family planning, global health, IUD, maternal health, postpartum

## Abstract

**Objective:**

The objective of this study was to compare postpartum hemoglobin (Hb) between postpartum intrauterine device (PPIUD) and non‐PPIUD users.

**Methods:**

A sample of 3697 postpartum women (475 PPIUD users, 3222 non‐PPIUD users) from 5 tertiary referral hospitals in Bangladesh were assessed at multiple time points between 6 weeks and 12 months postpartum. Non‐inferiority linear regression analysis compared changes in Hb levels at 29–52 weeks postpartum between the two groups. Non‐inferiority was declared if the lower 95% confidence interval of the estimated difference in Hb change since delivery between PPIUD and non‐PPIUD users was greater than −0.05 g/dl.

**Results:**

At approximately 9 months postpartum, 276 women in the PPIUD group (58.1%) and 1086 women in the comparison group (33.7%) attended follow‐up. In total, 57.9% of PPIUD users and 61.0% of non‐PPIUD users had taken iron supplementation. Change in Hb was 0.02 g/dl (95% CI: −0.16, 0.19) higher in the PPIUD users than the comparison group. The lower limit of the 95% CI was greater than −0.05 g/dl, providing good evidence that PPIUD users were non‐inferior to the comparison group in their Hb levels.

**Conclusion:**

In the presence of offering iron supplementation, and an uptake of just over 60%, no difference in anemia was observed between the PPIUD and control group.

## 1 INTRODUCTION

Iron deficiency anemia is a significant global health burden, affecting more than 1.5 million people worldwide.^1^ Defined as insufficient red blood cells to meet physiological needs, the diagnosis is based on hemoglobin levels and varies according to age, gender, altitude, smoking behaviors and different stages of pregnancy.^2^ In East Africa and South Asia, the prevalence of anemia among pregnant women is estimated to be 36% and 52%, respectively,^3^ and pooled data of demographic health surveys from seven South‐ and South‐East Asian countries has indicated a regional prevalence of anemia among women of reproductive age of 52.5%.^4^ In Bangladesh, the prevalence of anemia among women of reproductive age during the latest survey was slightly lower than this regional average, standing at 42%^5^; however, one study^6^ has suggested the prevalence of anemia among non‐pregnant, ever‐married women in Bangladesh to be much higher than levels reported in many other developed and developing countries around the world. Although rates of severe anemia are very low, the overall magnitude of the problem remains a major public health issue and rates are high enough to be of significant concern.^7^

Another significant public health challenge is the unmet need for contraception, which can lead to poor birth spacing and unintended pregnancies. In Bangladesh, the unmet need for a modern method of contraception among married/in union couples in 2019 was 20.1%, with an estimated 4 190 000 unintended pregnancies.^8^ Optimal provision of family planning services can prevent unintended pregnancies, reduce the number of women undergoing unsafe abortions, and ultimately reduce maternal mortality.^9^ Postpartum intrauterine device (PPIUD) is an innovative method of providing long‐acting reversible contraception (LARC) to women as a “one‐stop” procedure postpartum. If counseling regarding contraceptive methods is done in the antenatal period, and women choose the method, PPIUD can be inserted immediately after the birth of the baby and the placenta.^10^, ^11^, ^12^ The use of PPIUD has been shown to have few complications, to be highly effective at preventing a pregnancy, and is acceptable to women around the world.^13^, ^14^, ^15^, ^16^ The International Federation of Gynecology and Obstetrics (FIGO) PPIUD Initiative,^17^ in collaboration with the Obstetrical and Gynecological Society of Bangladesh (OGSB), allowed for comprehensive, non‐coercive, balanced discussions through designated counselors, who provided information on contraceptive methods and insertion of PPIUD for those choosing the method. In total, 186 394 women in Bangladesh received this service from September 2015 to March 2020.^18^, ^19^ The service proved to be highly acceptable to Bangladeshi women, with 9.3% (17296) choosing to receive a PPIUD. In the Government of Bangladesh Family Planning Costed Implementation Plan, commitments have been made both to increasing the uptake of LARC methods, such as copper IUDs, and to the promotion of postpartum contraception.^8^ The expectation is that in collaboration with OGSB, PPIUD will be offered at a national and sub‐national level through the government’s health service delivery system in the near future.^20^

Whilst copper intrauterine devices (Cu‐IUDs) are well established as a very effective method of contraception, some women using Cu‐IUDs may experience heavier, longer, and/or more painful periods. To date there have been very few studies on whether these menstrual changes result in an increased risk of anemia. Those studies that do exist have often been conducted on small samples or are now outdated.^21^, ^22^, ^23^ Furthermore, there are no studies looking at the impact of PPIUD use on the risk of developing anemia within the postpartum population. One study^24^ has reported increased lochia following PPIUD when comparing to non‐PPIUD users in Tanzania, but a link with anemia was not sought. Given the national intention to expand access to postpartum contraception and increase uptake of LARCs such as the copper IUD, this study aimed to increase the evidence base on the clinical implications of PPIUD use on hemoglobin levels as a proxy measure of anemia.

## 2 MATERIALS AND METHODS

This study was conducted as part of the FIGO PPIUD Initiative.^17^ The initiative operated in facilities across six countries (India, Sri Lanka, Bangladesh, Nepal, Tanzania, and Bangladesh) between 2013 and 2020, with the aim of institutionalizing the provision of PPIUD within routine family planning services. In Bangladesh, the initiative was active in six facilities, five of which served as sites for this research: Dhaka Medical College Hospital; Shaheed Suhrawardy Medical College Hospital; (Dhaka) Khulna Medical College Hospital; Sylhet Medical College Hospital; Chittagong Medical College Hospital.

Participant recruitment took place between April 7 to July 2, 2019, with all women attending the facilities for delivery being deemed eligible to participate. Women were provided with both written and verbal information in their local language prior to consenting to participate. Ethical clearance was provided by the Bangladesh Medical Research Council (BMRC/NREC/2016–2019/154). Recruited women completed an initial interview with a researcher in which basic demographic, health, and social data were recorded, and a hemoglobin reading was taken via finger prick blood sample and analyzed using a Hemocue 201+ (Mallinckrodt Inc.,). Iron supplementation was prescribed to all women and recorded for inclusion as a potential confounder during analysis. Information was also recorded on breastfeeding status and return of menses as potential confounding variables. Women were excluded from participating if their chosen method of contraception was hormonal implant, depot medroxyprogesterone acetate, or the combined oral contraceptive pill (COCP), due to the effect these contraceptives would have on menstruation. Recruitment continued across all sites until the minimum required sample size was reached. In total 475 PPIUD users and 3222 comparison group participants were recruited. As a higher proportion of PPIUD users were recruited from Khulna Medical College, analysis of key demographics across the sites using Chi square tests were conducted to ensure there were no significant differences between the two research groups.

Participants were requested to attend three follow‐up assessments, at 6 weeks, 6 months and 9 months postpartum. Between October 2019 and March 2020 all follow‐ups were conducted at facilities; however, due to the COVID‐19 outbreak, the study protocol was amended to enable researchers to complete follow‐ups through home‐visits. These were completed between August and September 2020. Participants from the PPIUD group were excluded if their IUD was expelled or removed after >7 days in situ. Participants were excluded from the comparison group if they subsequently took up a hormonal contraception method or interval IUD. Participants were excluded from both groups if a further pregnancy occurred.

Standardized compensation was provided to participants to cover the costs of transportation, and iron supplementation was provided free of charge. Follow‐up at all time periods included the administration of a standardized questionnaire collecting data on contraceptive use, iron supplementation, breastfeeding practice, and return of menses. In addition, those participants using PPIUDs were provided with a speculum examination at 6 weeks, in line with standard practice. At each follow‐up, participants were able to consult with a medical doctor and receive additional counseling and medical attention as required.

Data collection was completed using paper forms and later transferred to an online data collection platform (Commcare V.2.45.1; Dimagi^25^). Following data cleaning, statistical analysis was completed using Stata/IC 16.1 (StataCorp LLC.,). Follow‐up data were categorized by time since registration into four groups based on biological interest: 0–8 weeks inclusive (incorporating 6 week follow‐up); 9–28 weeks inclusive (incorporating 6 month follow‐up, which for breastfeeding women would have been a period of amenorrhea); 29–52 weeks inclusive (when menstruation is expected to have resumed); and >52 weeks (group followed up at home due to COVID‐19 restrictions). Mean Hb measurement and mean change in Hb since registration, and 95% confidence intervals, were reported for both groups at each categorical follow‐up time. The primary analysis was non‐inferiority in terms of Hb levels at 29–52 weeks postpartum. Where a woman attended two follow‐up visits within this time period, only the data collected closest to 36 weeks (9 months) postpartum were included in the non‐inferiority analysis. PPIUD would be considered non‐inferior in terms of Hb levels, relative to non‐PPIUD, if Hb levels among PPIUD users at 36 weeks postpartum were greater than the predefined non‐inferiority margin of −0.5 g/dl, i.e., women who received PPIUD might have a slightly smaller change in Hb since delivery than women without PPIUD, but the difference should be no more than 0.5 g/dl. Covariates identified a priori as potential confounders were included in all models: maternal age (centered at the median age of 25 years), parity, mode of delivery, breastfeeding, health facility, and baseline Hb measurement. Other covariates (return of menstruation, taking iron supplements) were investigated and included in the final model only if they improved the accuracy of the model, judged by reducing the standard error of the estimates.

Longitudinal analysis compared changes between the two groups in Hb over time from delivery (as a continuous variable) using a random effects linear regression model with a random intercept, including time as a linear and quadratic term.

## 3 RESULTS

In total, 3697 women were recruited immediately after delivery. Of these, 496 (13.4%) women had chosen a PPIUD, and the remaining 3201 (86.6%) had either declined any method or opted for an alternate non‐hormonal contraceptive. The demographics of the two groups were similar, except for the percentage of primiparous women (144 [29.0%] in PPIUD group, and, 1386 (43.3%) in comparison group) and differences in some categories of level of education and household income (Table 1).

**TABLE 1:** Baseline demographics of women recruited to each research group

		Baseline demographics	
		PPIUD group^a^	Comparison group^a^
Total women		496 (13.4)	3201 (86.6)
Categorical age	≤25	324 (65.3)	1990 (62.1)
	>25	172 (34.7)	1212 (37.9)
Education level	No education	38 (8.5)	355 (12.3)
	Primary	225 (50.2)	1221 (42.3)
	Secondary	155 (34.6)	1048 (36.3)
	Graduation/post‐graduation	30 (6.7)	262 (9.1)
Household income (BDT)	<5000	33 (6.7)	232 (7.2)
	5–10 000	244 (49.2)	1211 (37.8)
	10–15 000	138 (27.8)	915 (28.6)
	5–20 000	48 (9.7)	502 (15.7)
	>20 000	33 (6.7)	342 (10.7)
Mode of delivery	Vaginal delivery	310 (62.5)	2093 (65.4)
	Cesarean section	186 (37.5)	1109 (34.6)
Parity	Primiparous	144 (29.0)	1386 (43.3)
	Multiparous	352 (71.0)	1816 (56.7)

Abbreviations: BDT, Bangladeshi Takas.

^a^ Values expressed as number (percentage).

**FIGURE 1:**
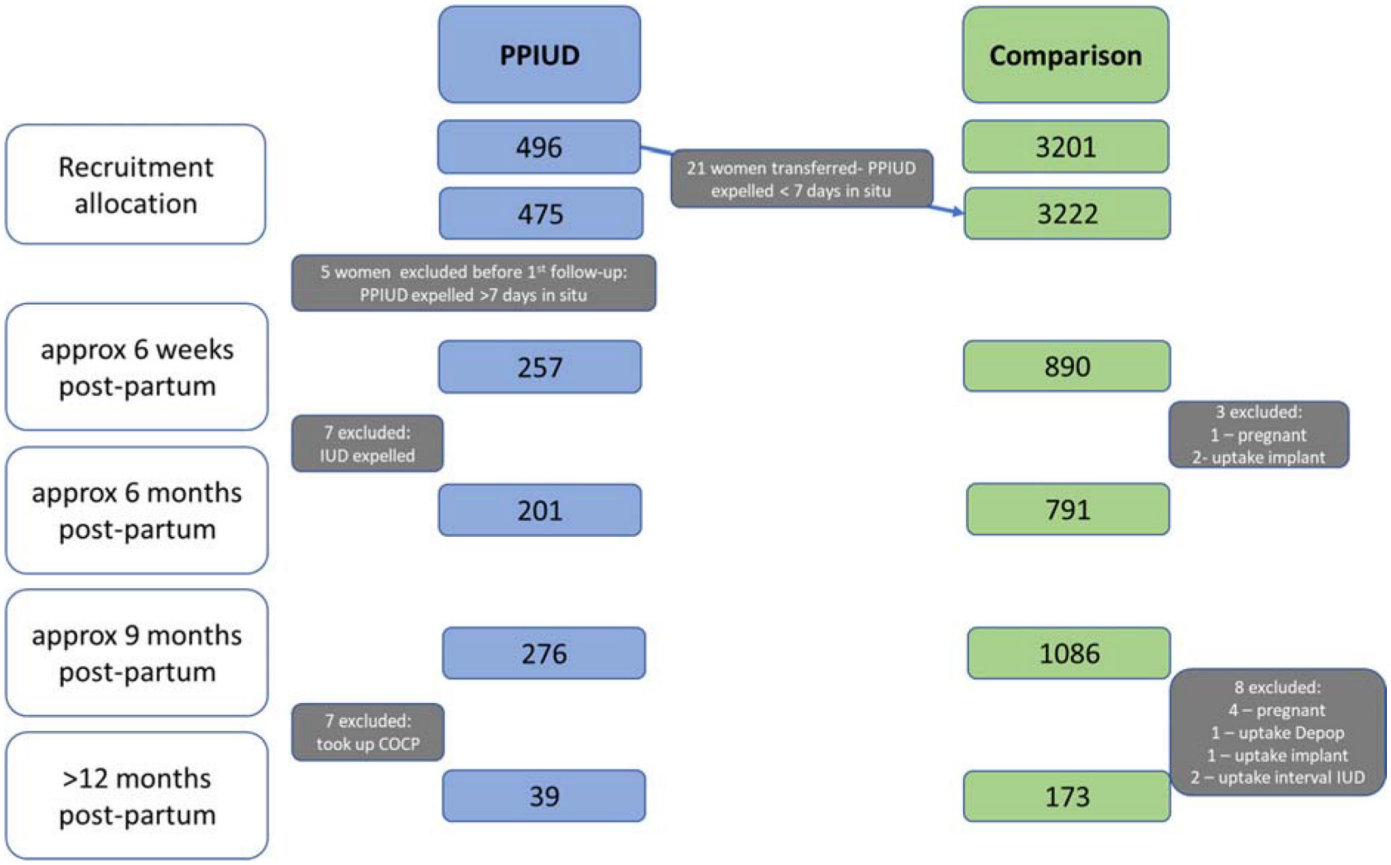
Flow diagram of group allocation

Before first follow‐up, 21 women were transferred from the PPIUD group to the comparison group due to the PPIUD being expelled within <7 days of insertion, and 5 women were excluded from the PPIUD group after recruitment but before attending any follow‐up visits due to the PPIUD being expelled >7 days after insertion. This resulted in 475 participants in the PPIUD group and 3222 in the comparison group. During the course of the study, 14 women from the PPIUD group were excluded: 7 due to the device being expelled or removed after more than 7 days in situ and 7 due to uptake of COCP. Follow‐up data collected on these participants prior to the point of exclusion were included in the analysis. Furthermore, 11 women were excluded from the comparison group during the course of the study due to either a further pregnancy or uptake of a hormonal contraceptive method, implant, or interval IUD. A breakdown of the number of participants in each research group and across the study is presented in Figure 1.

**FIGURE 2:**
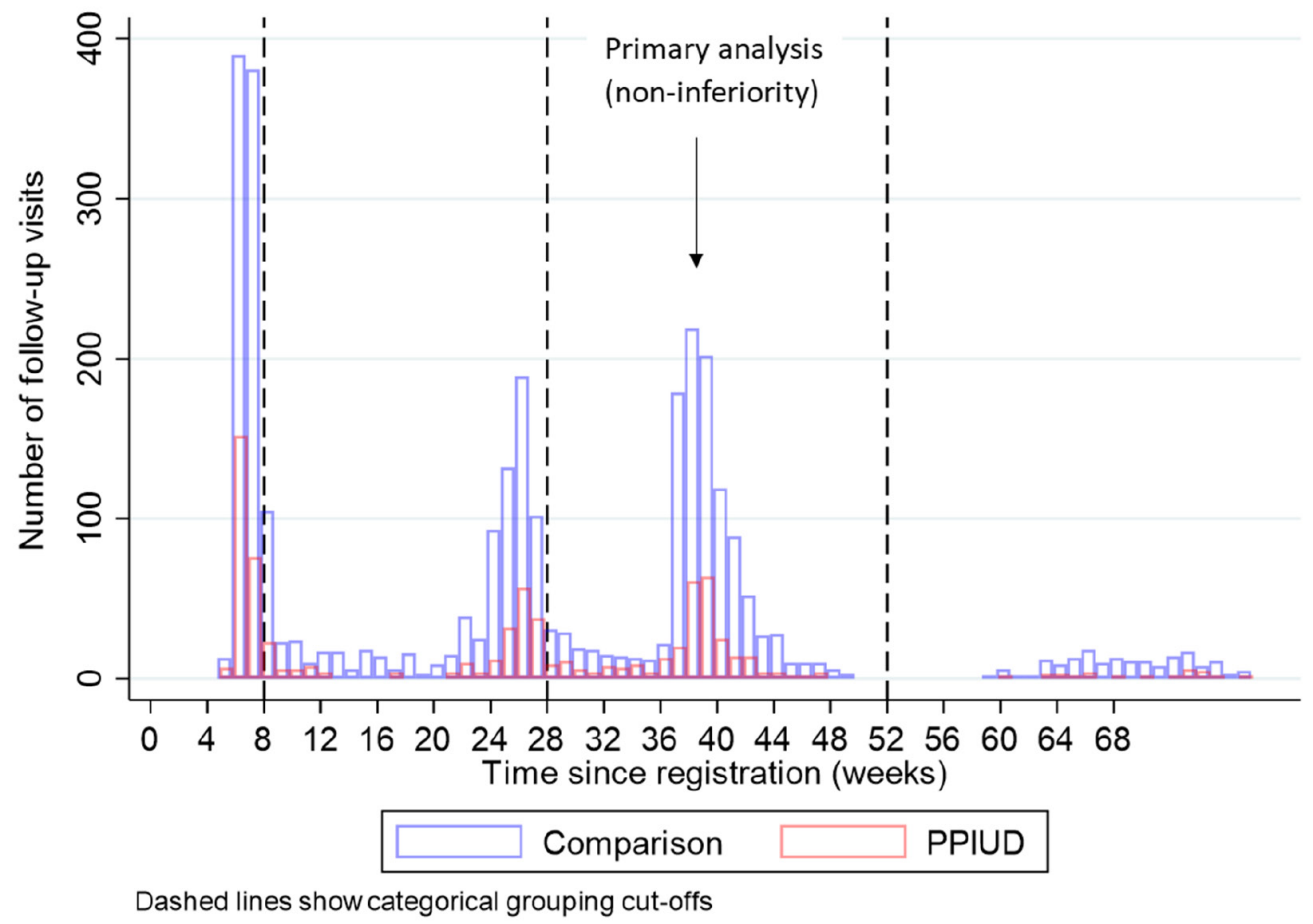
Distribution of follow‐up times by research group

**FIGURE 3:**
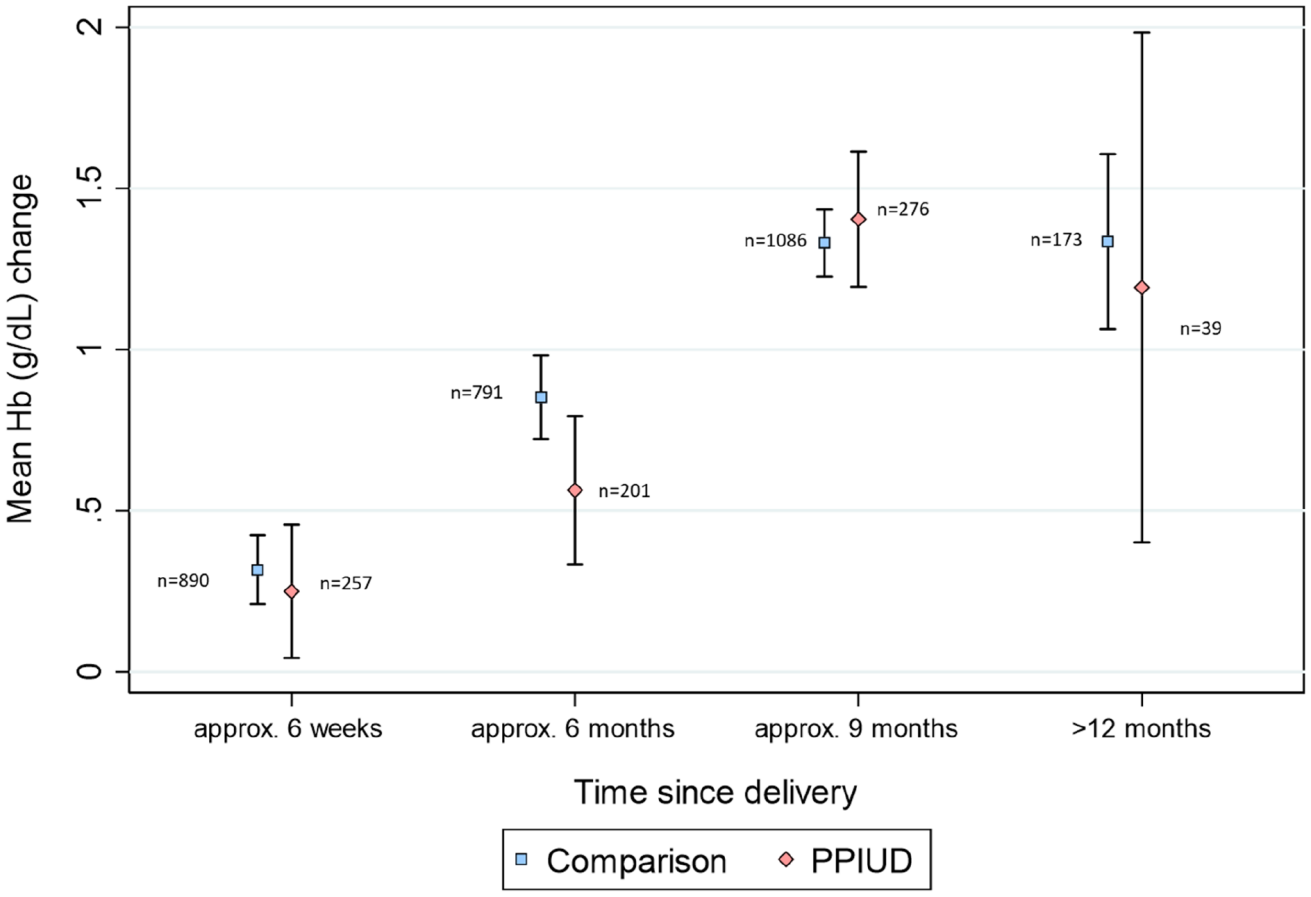
Mean change in Hb over time per time category and research group

The proportion of women attending follow‐up visits was consistently slightly higher in the PPIUD group than the comparison group (Table 2). Over the study period, a total of 130 women (27.4%) in the PPIUD group and 1807 (56.1%) women in the comparison group did not attend a single follow‐up visit. Attendance at all three follow‐ups was 189 (39.8%) among the women in the PPIUD group, and 614 (19.1%) among the comparison group. There were no statistically significant differences in characteristics between those who did and those who did not attend for follow‐up at the primary point of analysis (9 months) (Figures 2 and 3).

**TABLE 2:** Number of follow‐up visits attended by research group

Time point	Total (*n*)	%^a^	PPIUD group (*n*)	%^a^	Comparison group (*n*)	%^a^
Baseline	3697		475		3222	
6 weeks f/u^b^	1147	30.03	257	54.11	890	27.62
6 months f/u^b^	992	26.83	201	42.32	791	24.55
9 months f/u^b^	1362	36.84	276	58.11	1086	33.71
>12 months f/u^b^	212	5.73	39	8.21	173	5.37

Abbreviation: f/u, follow‐up.

^a^ Percentage of those registered.

^b^ Approximate time of follow‐up.

At the primary analysis point (~9 months), 276 (58.1%) women in the PPIUD group and 1086 (33.7%) women in the comparison group attended follow‐up. Among these women, there were no significant differences in terms of a priori confounders such as breastfeeding practice, return of menstruation, or use of iron supplementation. In total, 196 (83.7%) participants in the PPIUD group reported that they had resumed menstruation since delivery and 810 (79.6%) reported the same in the comparison group. Additionally, 136 (57.9%) women in the PPIUD group reported having taken iron supplements (tablets or injection) since delivery, compared to 618 (61.0%) women in the comparison group. Breastfeeding practices were also similar in both groups, with the majority having transitioned from exclusive breastfeeding to mixed feeding (223 [97.4%] in the PPIUD group vs. 896 [95.8%] in the comparison group) (Table 3).

**TABLE 3:** Comparison of a priori confounders between PPIUD and comparison groups at 9 month follow‐up

		9 month follow‐up		
		PPIUD group^a^	Comparison group^a^	*P* value
Menstruation returned		196 (83.7)	810 (79.6)	0.229
Taken iron supplements		136 (57.9)	618 (61.0)	0.376
Contraception method	BTL	0 (0.0)	143 (14.2)	<0.001
	LAM	0 (0.0)	1 (0.1)	
	POP & COCP	0 (0.0)	34 (3.4)	
	Condoms	0 (0.0)	645 (64.0)	
	Declined all methods	0 (0.0)	148 (14.7)	
	Other	0 (0.0)	13 (1.3)	
	Undecided	0 (0.0)	24(2.4)	
	PPIUD	234 (100.0)	0 (0.0)	
Breastfeeding	Formula/foods	1 (0.4)	9 (1.0)	0.701
	Exclusive breastfeeding	5 (2.2)	30 (3.2)	
	Breast and formula/foods	223 (97.4)	896 (95.8)	

Abbreviations: BTL, bilateral tubal ligation; COCP, combined oral contraceptive pill; LAM, lactational amenorrhea method, PPIUD; postpartum intrauterine device; POP, progestogen‐only pills.

^a^ Values expressed as number (percentage).

In both research groups mean Hb increased over time (Table 4). At the primary analysis time‐point (29–52 weeks), the increase in Hb was similar between groups, 1.40 (1.19, 1.61) g/dl in the PPIUD group and 1.33 (1.23, 1.44) g/dl in the comparison group (Table 4).

**TABLE 4:** Mean change in Hb by research group and follow up visit (95% CI)

	PPIUD			Comparison				
Time point	*n*	Mean Hb (95% CI)	Mean Hb change since registration (95% CI)^a^	*n*	Mean Hb (95% CI)	Mean Hb change since registration (95% CI)^a^	Difference in Hb change between groups (95% CI)^b^	*P* value
Registration	475	9.44 (9.30, 9.58)	—	3222	9.41 (9.36, 9.46)	—		
0–8 weeks	257	9.69 (9.52, 9.86)	0.25 (0.04, 0.46)	890	9.76 (9.68, 9.84)	0.32 (0.21, 0.42)	−0.07 (−0.30, 0.16)	0.542
9–28 weeks	201	9.91 (9.73, 10.08)	0.56 (0.33, 0.79)	791	10.20 (10.11, 10.30)	0.85 (0.72, 0.98)	−0.30 (−0.57, −0.02)	0.035
29–52 weeks	276	10.80 (10.65, 10.95)	1.40 (1.19, 1.61)	1086	10.84 (10.77, 10.92)	1.33 (1.23, 1.44)	0.08 (−0.14, 0.31)	0.473
>52 weeks	39	10.37 (9.86, 10.87)	1.19 (0.40, 1.98)	173	10.59 (10.35, 10.82)	1.33 (1.06, 1.61)	−0.15 (−0.83, 0.53)	0.663

*Note*. Nb. Negative values means Hb increase since registration was greater in comparison group than PPIUD group.

^a^ Positive value means an increase in Hb since registration.

^b^ Positive value means Hb increase since registration was greater in PPIUD than comparison group.

The primary non‐inferiority analysis was conducted on Hb data at 29–52 weeks postpartum. If a woman attended two follow‐up visits within this window, only the data collected closest to 36 weeks postpartum (9 months) were included. No potential confounders met the criteria for inclusion in the final model. Hb levels were 0.02 g/dl (−0.16, 0.19) higher in the PPIUD group than in the comparison group (*P* = 0.865), meaning that the increase in Hb was very slightly greater in the PPIUD group than the comparison group 29–52 weeks after delivery. The lower limit of the confidence interval of this difference between groups (−0.16) was greater than the pre‐defined non‐inferiority margin of −0.5 g/dl, providing good evidence that Hb levels in PPIUD users were non‐inferior to the comparison group.

To compare the change in Hb over time since delivery (i.e., not just at the 29–52 weeks non‐inferiority analysis time point) between the two groups, a random effects linear regression model was used, with time since delivery as a continuous variable. The square of the time variable was also included to allow for a non‐linear trend in Hb over time. The estimate of the difference in linear trend in Hb over time among the PPUID group relative to the comparison group was −0.001 (95% CI: −0.005, 0.002) g/dl per week (*P* = 0.786), providing no evidence of a difference in trend of Hb over time between the groups.

## 4 DISCUSSION

The present study set out to determine whether use of IUDs in the postpartum period presents any increased risk of anemia to women. This clinically important question was determined by measuring hemoglobin levels among both PPIUD users and demographically similar women using either no or non‐hormonal contraceptives within 12 months postpartum. Previous research in this area has been limited, with few studies investigating hemoglobin levels in copper IUD users and none focused specifically on the postpartum period. The research that does exist offers mixed findings, making it difficult to ascertain whether copper IUD use presents a true risk of anemia to women. A systematic review conducted in 2012, which focused on research reporting changes in hemoglobin levels of copper IUD users among women already diagnosed as anemic, identified 3 prospective cohort studies and 1 randomized control trial (RCT).^22^ The RCT trial and one of the three cohort trials revealed no statistical difference in hemoglobin levels, whilst the remaining two cohort trials demonstrated a statistically, but not clinically, significant difference. Further review of indirect evidence among non‐anemic women revealed 21 studies, including follow‐up to 5 years post‐insertion, which generally supported that there were no clinically significant changes.^22^

A further systematic review, also in 2012, specifically sought research on hemoglobin and serum ferritin levels among copper‐releasing and levonorgestrel‐releasing IUD (LNG‐IUS) users. This review identified 14 studies, 10 among non‐anemic and four among anemic women, as well as six studies of women using a LNG‐IUS. Meta‐analysis from these studies showed the LNG‐IUS resulted in an increase in hemoglobin levels, and whilst copper IUD users experienced statistically significant decreases, these were not clinically significant enough to induce anemia among previously non‐anemic women. Lowe et al.^23^ concluded that women who are borderline for a diagnosis of anemia may benefit from LNG‐IUS use. Neither of these studies, however, focused on the postpartum period, a critical period in which use of copper IUD presents an ideal opportunity for women who wish to delay or prevent further pregnancies, and who otherwise may have limited access to contraceptive services which are a safe and effective choice.

Participants from this study had a mean immediate postpartum Hb of <10 g/dl, the WHO threshold for postpartum anemia. This finding was higher than recently reported prevalence levels,^26^ which reviewed demographic health survey data and, using a classification for anemia of <10.9 g/dL, reported prevalence rates in Bangladesh of 46.3% in breastfeeding women and 56.4% in postpartum amenorrheic women. This paper identified women with postpartum amenorrhea to have approximately five times increased odds of having moderate to severe anemia (aOR = 4.81; 95% CI: 1.63–14.17; *P* = 0.004) and breastfeeding women to have slightly increased odds (aOR = 1.26; 95% CI: 1.01–1.57; *P* = 0.038) of mild anemia (11.0–11.9 g/dl). Other factors positively associated with increased odds were living in a rural area, and other demographics linked to living in poverty. Participants of our research were all recruited in urban hospitals; however, as these were tertiary referral centers, many of the population resided in rural areas, 50% had either no or only primary level education, and 47% reported an annual income of <10 000 BDT ($3.93 a day), which is only marginally higher than the World Bank poverty line for low‐ and middle‐income countries ($3.20 a day).^27^ It is possible that this accounts for the higher anemia rates observed within our cohort.

This study both analyzed hemoglobin levels as a continuous variable and assessed non‐inferiority at 9‐month follow‐up appointments. The results demonstrated that there is no statistically significant difference in the rate of hemoglobin recovery between PPIUD users and demographically similar non‐PPIUD postpartum women. We point out that all women in the study were offered iron supplementation with approximately a 60% uptake. Our study was designed to analyze hemoglobin levels over the first 9 months postpartum, but the COVID‐19 pandemic meant that we were unable to complete follow‐up of all women willing to attend at 9 months. Once lockdown protocols eased in the country, and we gained approval to conduct home follow‐ups using appropriate PPE, follow‐ups went ahead but with a 3‐month delay. On the positive side, this gave us extra insight into hemoglobin levels at >12 months postpartum, albeit of only 212 women (39 PPIUD and 173 non‐PPIUD). Although Hb levels were slightly lower in the PPIUD group when compared to the non‐PPIUD group, this difference was not found to be statistically significant. It would nevertheless be interesting in future studies to monitor Hb for a longer period postpartum as in contexts where the vast majority of women are exclusively breastfeeding, such as in Bangladesh, menstruation will only commence at 6 months once exclusive breast feeding ceases. This means that assessing Hb over a longer period would provide more long‐term evidence of its safety.

Our study provides important validation for the inclusion of copper IUDs as a method of contraception for women during this period, as it reduces the risk of unintended pregnancy and the wider health implications for both mother and baby. These findings are particularly relevant to low‐ and lower‐middle‐income settings where there is both a higher unmet need for contraception and burden of anemia. This research did not compare PPIUD use with hormonal long‐acting contraceptives such as LNG‐IUS, which research indicates may promote hemoglobin recovery through resulting long‐term amenorrhea,^28^ because these methods are not available in government facilities in Bangladesh. This is overwhelmingly the case in low‐ and middle‐income countries, albeit with isolated recent introduction of the hormonal IUS by donor‐funded international non‐governmental organization initiatives in some countries.^29^, ^30^, ^31^ This is primarily due to their prohibitive costs. Hormonal IUS was therefore not considered for this investigation, but it would be a highly relevant method were the cost addressed. Currently, the government of Bangladesh has committed to promoting postpartum contraception and increasing the uptake of LARCs through their FP2020 costed implementation plan (2020–2022), which includes PPIUD—our findings help to validate the safety of including PPIUD in these plans. The interval IUD and PPIUD are highly cost‐effective methods of contraception,^32^, ^33^, ^34^ and a model of a national‐level roll‐out of PPIUD services in Bangladesh was also found to be highly cost effective.^35^ With this evidence now dispelling concerns with regard to anemia, and its cost‐effective profile, the government can consider provision of postpartum family planning counseling with the inclusion of PPIUD as a very attractive public health policy.

In conclusion, this study was conducted in busy tertiary hospitals in a lower‐middle‐income country under real conditions. All women were offered iron supplementation with approximately a 60% uptake. Follow‐up rates at 9 months were 58% in the PPIUD group and 33% in the non‐PPIUD group. Under these conditions the study demonstrated that there was no evidence that PPIUD insertion was associated with anemia, or with a slower recovery of hemoglobin levels in the first 12 months postpartum. Offering women contraception in the postpartum period is an important public health measure for reducing unintended pregnancies and short birth spacing, both of which are associated with increased morbidity and mortality for mother and child. This study should provide reassurance to clinicians and family planning counselors who may otherwise have reservations about offering PPIUD to women who are at risk of anemia. However, further longer‐term studies would be advisable in order to reassure women who decide to continue the method for >12 months.

## AUTHOR CONTRIBUTIONS

AM conceived the idea for the research topic and together with SB, PF, FD and AY designed the study. SB and AY coordinated the data collection in country with support from AM, PF and FD. GGL provided ongoing epidemiological support and performed the statistical analysis. SB wrote the first draft with contributions from AM and GGL. All authors revised and approved the final version of the paper.

## CONFLICTS OF INTEREST

This research was conducted and funded as part of FIGO’s PPIUD initiative (2013–2020), which supported the institutionalization of comprehensive postpartum family planning counseling and PPIUD service provision into routine practice in Bangladesh, India, Sri Lanka, Nepal, Tanzania, and Kenya.

## Data Availability

The data that support the findings of this study are available on request from the corresponding author. The data are not publicly available due to privacy or ethical restrictions.
